# Measurement of Vital Signs Using Lifelight Remote Photoplethysmography: Results of the VISION-D and VISION-V Observational Studies

**DOI:** 10.2196/36340

**Published:** 2022-11-14

**Authors:** Emily Heiden, Tom Jones, Annika Brogaard Maczka, Melissa Kapoor, Milan Chauhan, Laura Wiffen, Helen Barham, Jeremy Holland, Manish Saxena, Simon Wegerif, Thomas Brown, Mitch Lomax, Heather Massey, Shahin Rostami, Laurence Pearce, Anoop Chauhan

**Affiliations:** 1 Portsmouth Hospitals University NHS Trust Portsmouth United Kingdom; 2 Mind Over Matter Medtech Ltd London United Kingdom; 3 The Text Doctor Wantage United Kingdom; 4 Xim Ltd Southampton United Kingdom; 5 Centre for Business Innovation Limited Cambridge United Kingdom; 6 Barts NIHR Biomedical Research Centre, Queen Mary University of London London United Kingdom; 7 School of Sport, Health & Exercise Science University of Portsmouth Portsmouth United Kingdom; 8 Data Science Lab, Polyra Limited Bournemouth United Kingdom

**Keywords:** general practice, vital signs/methods, vital signs/standards, photoplethysmography, remote photoplethysmography, remote photoplethysmography, Lifelight, contactless, software, algorithm development, algorithm, blood pressure, health monitoring, health technology, remote monitoring

## Abstract

**Background:**

The detection of early changes in vital signs (VSs) enables timely intervention; however, the measurement of VSs requires hands-on technical expertise and is often time-consuming. The contactless measurement of VSs is beneficial to prevent infection, such as during the COVID-19 pandemic. Lifelight is a novel software being developed to measure VSs by remote photoplethysmography based on video captures of the face via the integral camera on mobile phones and tablets. We report two early studies in the development of Lifelight.

**Objective:**

The objective of the Vital Sign Comparison Between Lifelight and Standard of Care: Development (VISION-D) study (NCT04763746) was to measure respiratory rate (RR), pulse rate (PR), and blood pressure (BP) simultaneously by using the current standard of care manual methods and the Lifelight software to iteratively refine the software algorithms. The objective of the Vital Sign Comparison Between Lifelight and Standard of Care: Validation (VISION-V) study (NCT03998098) was to validate the use of Lifelight software to accurately measure VSs.

**Methods:**

BP, PR, and RR were measured simultaneously using Lifelight, a sphygmomanometer (BP and PR), and the manual counting of RR. Accuracy performance targets for each VS were defined from a systematic literature review of the performance of state-of-the-art VSs technologies.

**Results:**

The VISION-D data set (17,233 measurements from 8585 participants) met the accuracy targets for RR (mean error 0.3, SD 3.6 vs target mean error 2.3, SD 5.0; n=7462), PR (mean error 0.3, SD 4.0 vs mean error 2.2, SD 9.2; n=10,214), and diastolic BP (mean error −0.4, SD 8.5 vs mean error 5.5, SD 8.9; n=8951); for systolic BP, the mean error target was met but not the SD (mean error 3.5, SD 16.8 vs mean error 6.7, SD 15.3; n=9233). Fitzpatrick skin type did not affect accuracy. The VISION-V data set (679 measurements from 127 participants) met all the standards: mean error −0.1, SD 3.4 for RR; mean error 1.4, SD 3.8 for PR; mean error 2.8, SD 14.5 for systolic BP; and mean error −0.3, SD 7.0 for diastolic BP.

**Conclusions:**

At this early stage in development, Lifelight demonstrates sufficient accuracy in the measurement of VSs to support certification for a Level 1 Conformité Européenne mark. As the use of Lifelight does not require specific training or equipment, the software is potentially useful for the contactless measurement of VSs by nonclinical staff in residential and home care settings. Work is continuing to enhance data collection and processing to achieve the robustness and accuracy required for routine clinical use.

**International Registered Report Identifier (IRRID):**

RR2-10.2196/14326

## Introduction

The regular measurement of vital signs (VSs) is an integral component of clinical care, as changes in VS often occur a few hours before an adverse event [1], providing an opportunity for intervention. However, the recording of VSs is often inadequate, such that clinical deterioration often goes unnoticed or is not detected in time to treat effectively [2]. In response to this challenge, the National Early Warning Score (NEWS) has been developed as a systematic approach to identify and respond to patients at risk of deterioration in health care settings based on the scoring of respiratory rate (RR), oxygen saturation, temperature, systolic blood pressure (SBP), pulse rate (PR), and level of consciousness [3]. The Recognise Early Soft Signs, Take Observations, Respond, Escalate (RESTORE2) system for use in care homes incorporates the NEWS alongside observations of soft signs to identify potential deterioration in clinical conditions [4]; however, this requires staff to be trained in the measurement of VSs.

VS measurement following discharge, for example, after surgery, is also important to identify deterioration. A European study of 193 readmitted patients identified marked deteriorations in PR (23%) and RR (28%) but only small changes (1%-2%) in blood pressure (BP) and oxygen saturation [5]. However, another study of 725 patients reported that, while 53% followed at least 10 of the recommended steps necessary for accurate BP measurement at home, only 1% followed all 15 recommendations [6]. Thus, home measurement of VSs is important—RR and PR in particular—but requires several pieces of equipment (BP monitor, pulse oximeter) and for patients to be educated in best practices.

Digital health technologies, such as wireless smart patches that measure PR and RR and finger clip BP monitors, have the potential to improve the ease and accuracy of VS measurement (Table 1). Photoplethysmography (PPG; the basis of pulse oximetry) enables the rapid and simultaneous measurement of VSs by detecting changes in the light reflected from the skin surface due to volumetric changes in the blood vessels. PPG has been used to measure PR [7,8], oxygen saturation [9], BP [10,11], and RR [7,12]. The COVID-19 pandemic has increased interest in using remote technology as a way to monitor patients with nonserious symptoms to reduce the burden on health care facilities, making them available for high-risk groups and the seriously affected, and to monitor patients with other medical conditions, thereby avoiding the risk of SARS-CoV-2 infection associated with visits to health care facilities [13]. Contactless technology is also potentially useful in situations where current care cannot be readily used, such as in mental health settings [14].

**Figure 1:**
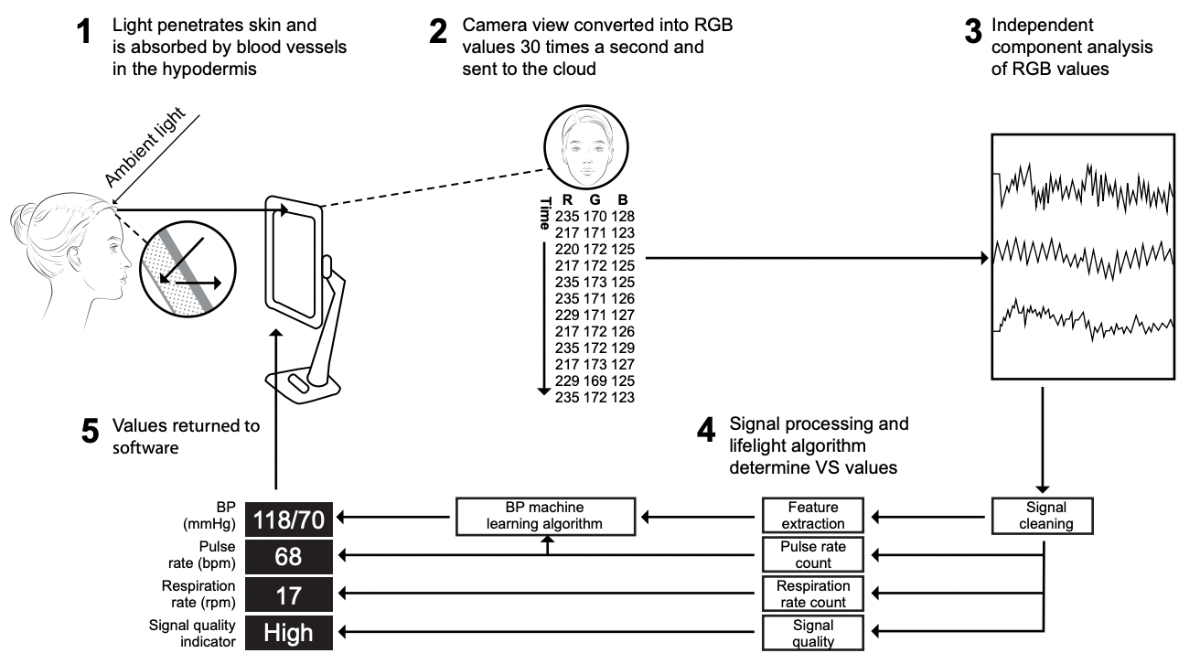
Use of remote photoplethysmography in the Lifelight software. BP: blood pressure; bpm: beats per minute; RGB: red, green, blue; rpm: respirations per minute; VS: vital sign.

Lifelight (Xim Ltd) is a novel software being developed as a medical device for the measurement of VSs by remote PPG (rPPG), based on live video capture of the face using the integral camera on smart devices (eg, laptops or smartphones). The software captures the average color of multiple regions of interest 30 times every second for 60 seconds; subtle changes in coloration are used to determine VSs (Figure 1).

**Table 1:** Accuracy performance targets for Lifelight.

Key innovative technology and vital sign			Target accuracy, mean error (SD)		Basis^a^		References
**Wireless smart patches**							
	Pulse rate (beats per minute)	2.2 (9.2)		Weighted average of performance of 3 devices		[15-17]	
	Respiratory rate (respirations per minute)	2.3 (5.0)		Weighted average of performance of 4 devices		[15,16,18,19]	
**Finger photoplethysmography monitor**							
	Systolic blood pressure (mmHg)	6.7 (15.3)		Weighted average of performance of 6 devices		[20-25]	
	Diastolic blood pressure (mmHg)	5.5 (8.9)		Weighted average of performance of 6 devices		[20-25]	

^a^Relevance and quality scores: 3.0-3.16 for pulse rate, 2.83-3.16 for respiratory rate, 2.83-3.5 for systolic/diastolic blood pressure.

Here, we report early validation steps of the Lifelight software for the measurement of PR, RR, SBP, and diastolic BP (DBP; in line with the intended purpose of Lifelight) [26]. As Lifelight is a contactless technology, there are no relevant International Organization for Standardization (ISO) standards for validation. The most similar devices are wireless smart patches and finger clip BP monitors. We therefore developed a novel methodology for validation, informed by the standards for pulse oximetry [27] and noninvasive sphygmomanometers [28], and a validation study of the pyroelectric polymer for measuring RR [29]. A rigorous systematic literature review was performed according to the PRISMA (Preferred Reporting Items for Systematic Reviews and Meta-Analyses) guidelines to identify the performance of relevant devices for each VS. Quality and relevance scores were used to weight the findings (average score out of 4 for methodological quality: study design, sample size, method of comparison; scientific validity; and relevance to Lifelight’s intended purpose). These performance targets (Table 1) were calculated in preparation for a Conformité Européenne (CE)–marking audit. As per criterion 1 of the standard for noninvasive sphygmomanometers [28], the mean error and SD of the Lifelight measurements are compared with standard of care (SOC) measurements recorded concurrently. The targets in Table 1 have been approved by the UK Health Research Authority (HRA) for the ongoing Vital Sign Comparison Between Lifelight and Standard of Care (VISION) Acute study (NCT04589923).

Here, we report two early studies in the development of Lifelight. The objective of the Vital Sign Comparison Between Lifelight and Standard of Care: Development (VISION-D) study (NCT04763746) was to collect RR, PR, and BP measurements simultaneously by using the current SOC manual methods and the Lifelight software to iteratively refine the software algorithms. The objective of the Vital Sign Comparison Between Lifelight and Standard of Care: Validation (VISION-V) study (NCT03998098) was to validate the use of Lifelight software to accurately measure VSs.

## Methods

### VISION-D

VISION-D was a prospective observational study conducted over 12 months during 2018 and 2019 [26], involving 8585 inpatients, outpatients, and healthy volunteers aged >3 years. There were no exclusion criteria to ensure local representation in age, sex, health condition, and skin tone and the inclusion of a wide range of VS values within and outside normal healthy ranges. The sample size was expected to exceed 2000 volunteers but was not formally prespecified, as it would depend on the incremental improvement in accuracy of the Lifelight system. The study continued until acceptable accuracy was achieved through machine learning. The sponsor kept the study teams informed on progress.

The study was conducted at Queen Alexandra Hospital, Portsmouth Hospitals University National Health Service (NHS) Trust in accordance with Good Clinical Practice and was approved by the HRA (Integrated Research Application System number: 242581). All participants gave written informed consent.

**Figure 2:**
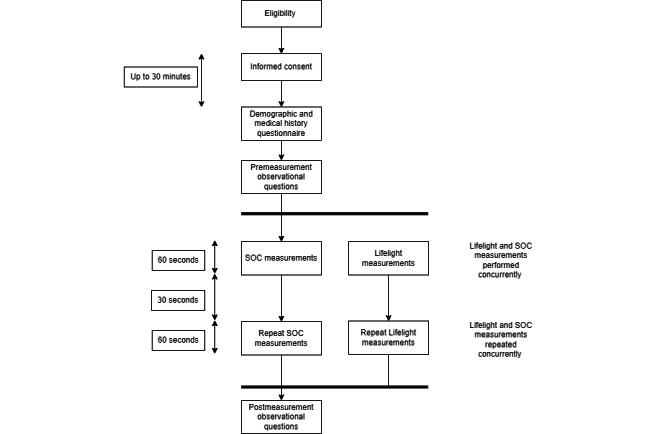
Data collection in VISION-D. SOC: standard of care; VISION-D: Vital Sign Comparison Between Lifelight and Standard of Care: Development.

Measurements were taken by trained nursing staff and clinical trial assistants. PR and BP were measured with a standard clinical automatic sphygmomanometer (Welch Allyn Connex Spot Monitor) on one arm, allowing both to be measured simultaneously, rather than also using an electrocardiogram to record PR. RR was determined via the manual counting of observed inspirations over 60 seconds. The Lifelight software was run on a sixth generation Apple iPad, held approximately 1 meter from the participant and angled toward their face. Measurement started and stopped automatically, and the data were sent to a secure database without being displayed (to prevent clinical interpretation or analysis). Two sets of measurements were taken by two staff members during the same 60-second period and then repeated, giving 4 sets in total (Figure 2). Pre- and postmeasurement observations were made of background luminosity, temperature, the use of makeup, and facial features.

Transmitted data were encrypted and stored in a secure database. No identifiable data were stored. Only data for adults (≥18 years) were reported. The data were used to train the software algorithms: the ensemble machine learning algorithm Extra Trees [30] was used for BP, and the filtering of the Fourier-transformed space followed by shape feature counting was used for PR and RR.

### VISION-V

**Figure 3:**
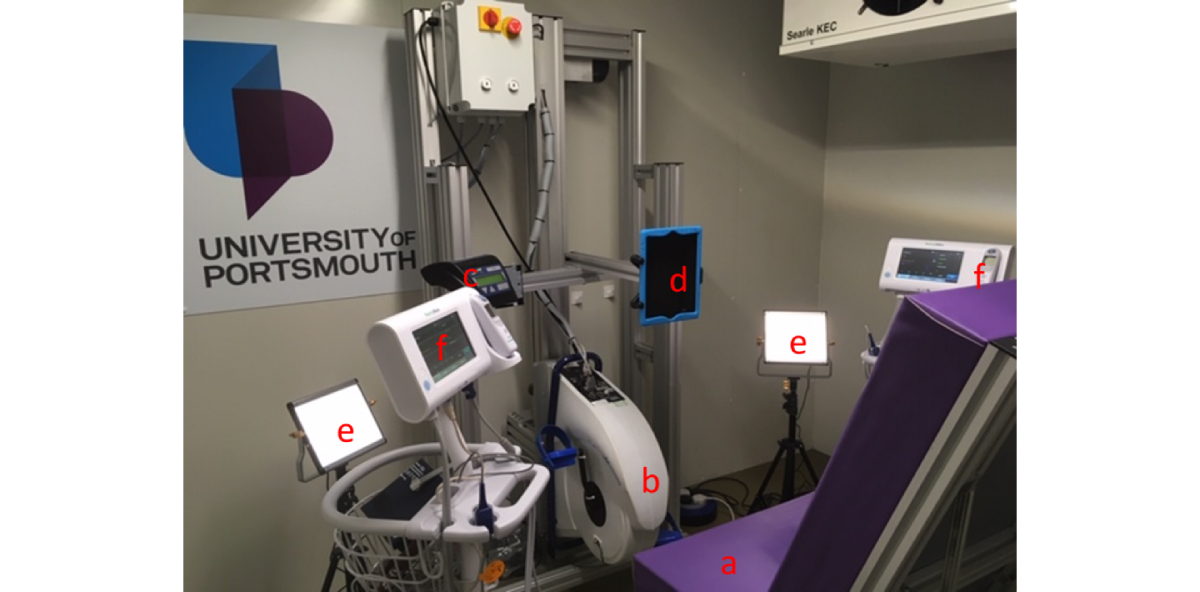
The environmental chamber used for hypoxic exercise testing and normoxic blood pressure (BP) evaluation: (a) recumbent chair; (b) ergometer; (c) ergometer control panel used for cycle exercise; (d) iPad running Lifelight software; (e) photographic lights to supplement chamber lighting; (f) Welch Allyn Connex Spot Monitors used for measuring BP, pulse rate, and oxygen saturation.

VISION-V (n=127) was conducted at the School of Sport, Health and Exercise Science at the University of Portsmouth, United Kingdom, during 2019. Measurements were performed as in VISION-D but in a normobaric hypoxic chamber (Figure 3)*.* VSs were measured 3 times in each participant by two observers who were blinded to their device readings and to each other’s readings. Data collection was overseen by an independent supervisor. The study was conducted in accordance with Good Clinical Practice and approved by the HRA (Integrated Research Application System number: 258187). All participants gave written informed consent.

In addition to standard VSs measurement, healthy participants aged 18-39 years exercised on a recumbent cycle ergometer (maximum intensity 200 W) to generate a wide range of PR and RR values, per the laboratory’s standard operating procedure and under the advice of the independent medical officer. The exercise intensity and hypoxic environment were individually titrated to induce ≥80% oxygen desaturation. VSs were measured immediately after each exercise bout.

### Ethics Approval

Ethical approval for VISION-V was granted by the London-Dulwich Research Ethics Committee (reference 19/LO/0427). The Medicines and Healthcare products Regulatory Agency issued a notice of no objection for the medical device to be used in VISION-V (reference CI/2018/0078). VISION-D was approved by the HRA and Health and Care Research Wales (reference 18-NS-0047). All participants provided written informed consent.

### Statistical Analysis

In VISION-D, the enrolled set comprised all recruited participants; the full analysis set (FAS) comprised those for whom VS measurements are included. Reasons for exclusion were an age of <18 years, incorrect or incomplete data entry, physiologically implausible data (determined by the clinical investigator), and low signal quality (pulse signal quality indicator <0.85; eg, because of excessive movement or insufficient light).

To ensure the accuracy of sphygmomanometers over a clinically useful range, the ISO standard for SBP requires that ≥5% of measurements are ≤100 mmHg, ≥5% are ≥160 mmHg, and ≥20% are ≥140 mmHg [28]. For DBP, ≥5% of measurements should each be ≤60 mmHg and ≥100 mmHg, and ≥20% should be ≥85 mmHg [28]. We therefore analyzed similar BP subgroups constructed using data randomly selected from the full data set; the distribution was calculated by up-weighting all SBP/DBP bands not meeting the minimum percentages to become ≥5% or ≥25% of the subgroup as appropriate and down-weighting bands exceeding the minimum percentages.

A subgroup was also created using the Fitzpatrick Skin Type Scale [31], comprising ≥5% each in groups 1 and 4-6 and ≥20% each for groups 2 and 3, with up- and down-weighting as described for the BP subgroup.

The primary analysis in both studies assessed the performance of Lifelight against the SOC measurements; an accuracy target was deemed to be met if mean error and SD for Lifelight measurements at least equaled the target (Table 1). Heat maps were generated for the VISION-D data, as the large amount of data rendered a scatter plot unclear. Scatter plots were developed for the smaller VISION-V data set (which was insufficient for a heat map).

Linear regression was used to assess the impact of skin tone on the accuracy of Lifelight for measuring each VS, using the Fitzpatrick skin tones as the exploratory variable.

## Results

### VISION-D

The enrolled set comprised 8585 participants; 60%-67% were included in individual VS analyses, and 17,233 measurements were collected, of which 43%-59% were included in the individual VS analyses (FAS). Demographic details are provided in Table 2. There were no protocol deviations or adverse events.

**Figure 4:**
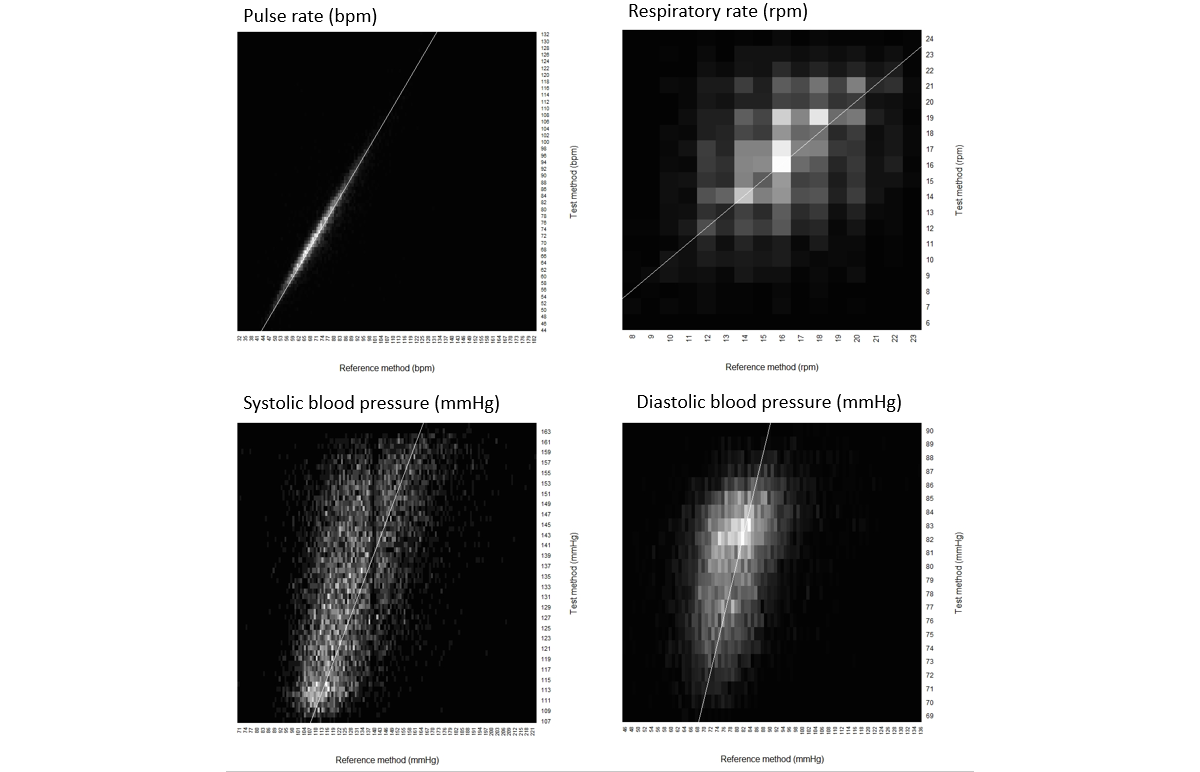
Heat maps for correlation between Lifelight (test methods) and standard of care (reference) measurements in VISION-D. The density of white points illustrates the extent of overlap. Correlation coefficients (R^2^) were 0.89 for pulse rate, 0.11 for respiratory rate, 0.30 for systolic blood pressure, and 0.15 for diastolic blood pressure. The line of identity (y=x) illustrates when the Lifelight (test) measurement provided was the same as the reference. bpm: beats per minute; VISION-D: Vital Sign Comparison Between Lifelight and Standard of Care: Development.

The performance targets were met for all measurements except SBP in the FAS and the BP subgroup (Table 3). Heat maps of the reference method (SOC manual measurement) versus the test measurement are shown in Figure 4. Values for RR fell within a narrow range, distorting the appearance of the heat map. Amplifying the proportion of DBP data at extreme values slightly reduced the accuracy whereas amplifying the proportion of SBP data at extreme values had little effect (analysis not shown).

**Table 2:** Characteristics of the final analysis set in VISION-D^a^.

Characteristic			Participants^b^	Value, range		Value, mean (SD)	
**Sex**					N/A^c^		N/A
	Female, n (%)		5649 (65.8)	N/A^c^		N/A	
	Male, n (%)		2936 (34.2)	N/A		N/A	
Age (years), n			8585	4-96		49.7 (17.1)	
**Fitzpatrick skin tone, n (%)^d^**					N/A		N/A
	**1**						
		RR^e^	197 (3.44)				
		PR^f^	170 (3.30)				
		SBP^g^	158 (2.98)				
		DBP^h^	149 (2.89)				
	**2**						
		RR	2967 (51.81)				
		PR	2646 (51.41)				
		SBP	2733 (51.54)				
		DBP	2663 (51.69)				
	**3**						
		RR	2292 (40.02)				
		PR	2068 (40.18)				
		SBP	2162 (40.77)				
		DBP	2101 (40.78)				
	**4**						
		RR	189 (3.30)				
		PR	185 (3.59)				
		SBP	175 (3.30)				
		DBP	169 (3.28)				
	**5**						
		RR	28 (0.49)				
		PR	29 (0.56)				
		SBP	23 (0.43)				
		DBP	22 (0.43)				
	**6**						
		RR	5 (0.09)				
		PR	2 (0.04)				
		SBP	5 (0.09)				
		DBP	5 (0.10)				
	**Unassigned**						
		RR	49 (0.86)				
		PR	47 (0.91)				
		SBP	50 (0.94)				
		DBP	43 (0.83)				
PR (beats per minute)			5727	32-183		73.6 (12.2)	
RR (respirations per minute)			5147	8-23		16.1 (2.8)	
SBP (mmHg)			5303	71-223		130.7 (19.4)	
DBP (mmHg)			5152	46-136		79.9 (9.2)	

^a^VISION-D: Vital Sign Comparison Between Lifelight and Standard of Care: Development.

^b^Values for sex and age data are for the enrolled set; data for vital signs are for the full analysis set; only data from adults (>18 years) were analyzed.

^c^N/A: not applicable.

^d^Not all measurements contributed to each vital sign (eg, not everyone with a Fitzpatrick skin tone of 1 had respiratory rate, pulse rate, systolic blood pressure, and diastolic blood pressure analyzed because of exclusions, such as poor signal quality).

^e^RR: respiratory rate.

^f^PR: pulse rate.

^g^SBP: systolic blood pressure.

^h^DBP: diastolic blood pressure.

**Table 3:** Performance of Lifelight in VISION-D^a^.

	Measurements (n=17,233), n (%)	Accuracy, mean error (SD)		
		Target	Full analysis set	BP^b^ subgroup
Pulse rate (beats per minute)	10,214 (59)	2.2 (9.2)	0.3 (4.0)	N/A^c^
Respiratory rate (respirations per minute)	7462 (43)	2.3 (5.0)	0.3 (3.6)	N/A
Systolic BP (mmHg)	9233 (54)	6.7 (15.3)	3.5 (16.8)	4.1 (17.0)
Diastolic BP (mmHg)	8951 (52)	5.5 (8.9)	−0.4 (8.5)	−1.0 (10.0)

^a^VISION-D: Vital Sign Comparison Between Lifelight and Standard of Care: Development.

^b^BP: blood pressure.

^c^N/A: not applicable.

For the Fitzpatrick subgroup, the performance targets were met for PR, RR, and DBP; for SBP, the standard was met for mean error but not SD (Table 4). The regression analysis for skin tone showed only small changes in error between one Fitzpatrick group and the next, with similar changes in error for the FAS and Fitzpatrick subgroup (Table 5).

As data accumulated, signal processing was used to improve the accuracy of PR and RR measurement and machine learning for BP. The SBP SD decreased from 22 to 14 mmHg over the 12-month study. The proportion of measurements ≤5 mmHg of the reference doubled from 15% to 30%, and the percentage of measurements ≤10 mmHg increased from 30% to 50%.

**Table 4:** Performance of Lifelight in VISION-D^a^ in the Fitzpatrick subgroup.^b^

	Eligible measurements, n	Target, mean error (SD)	Accuracy, mean error (SD)
Pulse rate (beats per minute)	6700	2.2 (9.2)	0.3 (4.0)
Respiratory rate (respirations per minute)	4520	2.3 (5.0)	0.4 (3.7)
Systolic blood pressure (mmHg)	5152	6.7 (15.3)	3.6 (16.6)
Diastolic blood pressure (mmHg)	4960	5.5 (8.9)	−0.4 (8.5)

^a^VISION-D: Vital Sign Comparison Between Lifelight and Standard of Care: Development.

^b^The subgroup comprised ≥5% each in groups 1 and 4-6 and ≥20% each for groups 2 and 3.

**Table 5:** Regression analysis for skin tone.

	Full analysis set			Fitzpatrick subgroup	
	Measurements, n	Change in error^a^	Measurements, n		Change in error
Pulse rate (beats per minute)	10,131	0.45	6700		0.46
Respiratory rate (respirations per minute)	7391	0.11	4520		0.13
Systolic blood pressure (mmHg)	9148	0.5	5152		1.1
Diastolic blood pressure (mmHg)	8870	−0.8	4960		−0.8

^a^Values are the change in error from one Fitzpatrick group to the next in the full analysis set and the Fitpatrick subgroup, comprising ≥5% each in groups 1 and 4-6 and ≥20% each for groups 2 and 3.

### VISION-V

**Figure 5:**
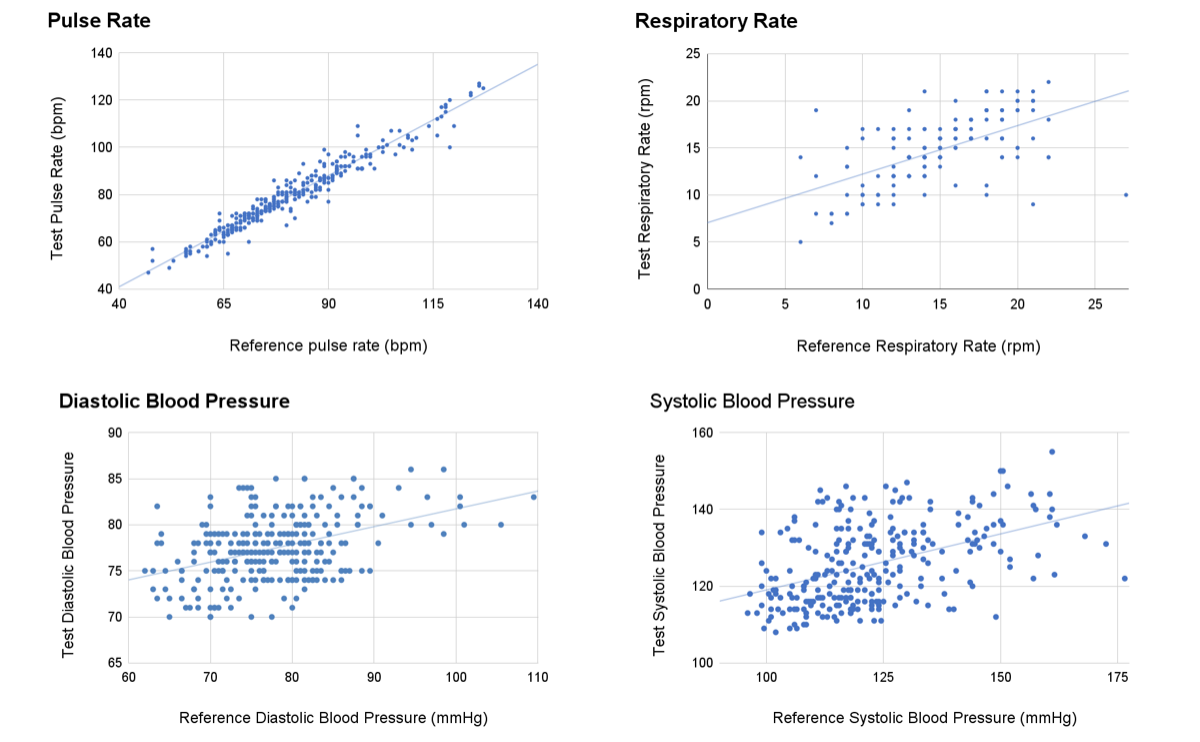
Scatter plots with correlation lines for the vital signs measured with Lifelight (test) versus standard of care (reference) method. Correlation coefficients (R2) were 0.94 for pulse rate (PR), 0.30 for respiratory rate (RR), 0.21 for systolic blood pressure (SBP), and 0.17 for diastolic blood pressure (DBP); 95% Bland-Altman limits of agreement were −5.9 and 8.8 bpm for PR, −6.9 and 6.6 for RR, −25.6 and 31.2 for SBP, and −14.1 and 13.4 for DBP. bpm: beats per minute; rpm: respirations per minute.

Characteristics of the FAS (n=125) are presented in Table 6. There were no protocol deviations or adverse events. For the different VSs, 61%-83% of measurements were eligible for the performance analysis (Table 7). The scatter plots showed good correlations between Lifelight and SOC measurements of VSs (Figure 5). The performance targets for the FAS were met for all VSs (Table 8).

**Table 6:** Reference data for the full analysis set in VISION-V^a^ (n=125 participants).

Characteristic			Value		Value, mean (SD)
**Sex, n (%)**					N/A^b^
	Male	55 (44)			
	Female	70 (56)			
Age (years), range			18-66		30.2 (11.6)
Pulse rate (beats per minute), range			47-127		79.4 (15.2)
Respiratory rate (respirations per minute), range			6-27		14.8 (3.9)
Systolic blood pressure (mmHg), range			96-176.5		122.9 (15.9)
Diastolic blood pressure (mmHg), range			62-109.5		77.8 (7.8)

^a^VISION-V: Vital Sign Comparison Between Lifelight and Standard of Care: Validation.

^b^N/A: not applicable.

**Table 7:** Eligible measurements in VISION-V^a^.

	Participants included in analyses, n	Eligible measurements^b^, n (%)
Pulse rate	72	308 (83)
Respiratory rate	67	143 (61)
Systolic blood pressure	115	115 (69)
Diastolic blood pressure	113	113 (64)

^a^VISION-V: Vital Sign Comparison Between Lifelight and Standard of Care: Validation.

^b^Complete measurement sets where the photoplethysmography signal quality was adequate to measure vital signs using Lifelight. Lifelight measurements were eligible for the analysis if the photoplethysmography signal quality was ≥0.85 and the measurement set was complete (one set each for pulse rate, systolic blood pressure, and diastolic blood pressure.

**Table 8:** Accuracy of Lifelight in VISION-V^a^.

	Target accuracy, mean error (SD)	Accuracy in VISION-V, mean error (SD)
Pulse rate (beats per minute)	2.2 (9.2)	1.4 (3.8)
Respiratory rate (respirations per minute)	2.3 (5.0)	−0.1 (3.4)
Systolic blood pressure (mmHg)	6.7 (15.3)	2.8 (14.5)
Diastolic blood pressure (mmHg)	5.5 (8.9)	–0.3 (7.0)

^a^VISION-V: Vital Sign Comparison Between Lifelight and Standard of Care: Validation.

## Discussion

### Principal Results

VISION-D and VISION-V demonstrate the accuracy of the Lifelight software in the simultaneous contactless measurement of VSs, based on more than 17,000 measurements. The predefined performance targets were met for PR, RR, and DBP in VISION-D; for SBP, the mean error was met but not SD (Table 3). All targets were met in VISION-V (Table 8). On the basis of these data, Lifelight achieved Level 1 CE mark certification as a medical device [14].

The use of mobile devices for measurement of VSs presents some challenges compared with controlled laboratory scenarios, for example [32]. To mitigate some of these challenges, we have compared VSs that were measured simultaneously by using SOC methods and Lifelight. We believe VISION-D to be the largest study to date to measure VSs using rPPG. As there are currently no standards for contactless measurement of VSs, we developed performance targets in discussion with the HRA and a CE-marking auditor (Table 1). The HRA has accepted these targets for the ongoing VISION-Acute study (NCT04589923), and the targets can therefore be considered applicable to the VISION-V and VISION-D studies. The software algorithms were refined continuously by using data collected during VISION-D, and the final algorithms were used in VISION-V. The accuracy targets (set before data analysis) were met in VISION-V. Although participants in VISION-V had a wide range of VS values, the ISO distribution criteria for BP were not met, likely because the participants were from a healthy population (few had hypotension or hypertension). However, amplifying the proportion of participants with high/low BP in VISION-D did not affect the accuracy of SBP measurement, but the SD for the DBP performance target was no longer met.

Although the accuracy targets were met for RR, values recorded by Lifelight were 10 to 20 respirations per minute (rpm), whereas the reference values were 5 to 22 rpm, indicating that there may be some loss of accuracy at the slower rates. An RR above 22 rpm is clinically important but was not captured in the VISION-V and VISION-D studies, likely because the participants were mostly healthy. This is being addressed in both the VISION-Acute and the VISION: Multisite Development (VISION-MD) studies (NCT04589923 and NCT04763746, respectively), which are enrolling a broader range of patients with VS values outside the normal range, including some who are critically ill, to improve the accuracy of Lifelight for clinical use.

Our substantial database from VISION-D includes medical history, temperature, light (lux meter), Fitzpatrick skin tone, facial tattoos, birthmarks, facial hair, etc, which can be used to explore potential interference factors (in contrast to the Medical Information Mart for Intensive Care PPG database of patients who are critically ill [33]).

### Limitations and Future Work

Studies in 2018 and 2019 demonstrated the potential of PPG to detect changes in cardiovascular activity and the measurement of BP [34-36]; a recent study claims to meet the ISO standards for BP measurement (ISO 81060-2), based on 225 measurements in 85 volunteers [37]. The accuracy in VISION-V was also within the ISO 81060-2 standard (5 ± 8 mmHg) for DBP, and it was within the mean error but not within the SD for SBP, although this ISO relates to the cuff-based measurement of BP. In addition, based on the mean error in VISION-D, the performance of Lifelight was comparable to that reported in the literature for most of the devices on which the standards were based. BP is inherently more complex to measure than PR and RR, in terms of the data form and machine learning and because reference measurements are less accurate.

As with any recording device, signal quality may be compromised if the participant moves excessively or light levels are insufficient. The proportion of eligible measurements ranged from 61% for RR to 83% for PR. Ineligible measurements were largely due to the inadequate quality (blurring) of the video recordings. Higher-resolution video recording is being used in the current VISION studies (described in more detail below), which is expected to provide a cleaner and more robust signal. However, Lifelight is easy to use, and measurements can be repeated within a minute in the event of a poor signal.

Skin type is a potential source of error with PPG devices, as melanin absorbs green light, potentially increasing errors in measurements in dark-skinned individuals compared with light-skinned individuals [38]. However, skin type does not affect the accuracy of Lifelight: the performance targets were met for RR, PR, and DBP in the Fitzpatrick subgroup, and mean error was met for SBP but not SD. Moreover, amplifying the proportion of participants with light and dark tones did not affect accuracy. Bent and colleagues [38] also reported that Fitzpatrick skin type had no significant effect on the accuracy of PR measurements by wearable optical heart rate sensors; however, this was a small study (n=53). Although the Fitzpatrick Skin Type Scale is the current gold standard [31], its use has been criticized because of racial bias, weak correlation with skin color, and broad within-group variations in skin tone. Spectrocolorimetry, which uses multiple variables to categorize skin tone objectively, has been proposed as an alternative [31], which may be incorporated into later studies to confirm our findings.

The accuracy of the Lifelight algorithms will further improve with continuing data collection. The ongoing VISION-MD study is collecting data from a wide range of participants, including patients who are critically ill, which will be used for algorithm development and then testing. Higher-resolution video data are being collected in this study, and the algorithms are focusing on smaller but higher-quality regions of interest.

### Comparison With Prior Work

The use of rPPG offers several advantages in addition to the rapid and contactless measurement of RR, PR, and BP simultaneously. There is no need for calibration, servicing, cleaning several pieces of equipment, or specialist training. Such advantages are particularly useful in residential care. Indeed, Lifelight has been piloted with the Hampshire Hospitals NHS Foundation Trust as part of a telemedicine service during the COVID-19 pandemic [39]. Care teams found the software easy to use and care was improved, as residents did not need to travel and VSs could be recorded easily by a known carer; clinicians’ travelling time was also reduced. Another study of remote VS monitoring in residential care reported that 87% of emergency department attendances were avoided [40]. Less tangible but valuable benefits include reduced anxiety among staff and residents, particularly the fear of hospitalization [40].

Notably, RR is often missed from VS monitoring or measured inaccurately [41], but changes in RR can be a harbinger of physiological conditions such as hypoxia, hypercapnia, and acidosis [42]. During the COVID-19 pandemic, patients at risk in England were provided with pulse oximeters, with instructions to seek medical help if oxygen saturation fell below 92% [43]. However, changes to RR indicate increased ventilation and precede reductions in oxygen saturation [41], thus giving an earlier indication of clinical deterioration. A PPG device to record RR would therefore be invaluable in this situation. The COVID-19 pandemic also highlighted the importance of contactless VS measurement [13].

### Conclusion

This preliminary evaluation of Lifelight demonstrates sufficient accuracy in the measurement of VSs to support Level 1 CE mark certification, with further work ongoing to develop Lifelight into a robust method for measurement of VSs in daily clinical use. As the use of Lifelight does not require specific training or equipment, the software is potentially useful for the contactless measurement of VSs by nonclinical staff in residential and home care settings.

## Abbreviations

BP: blood pressure
CE: Conformité Européenne
DBP: diastolic blood pressure
FAS: full analysis set
HRA: Health Research Authority
ISO: International Organization for Standardization
NEWS: National Early Warning Score
NHS: National Health Service
PPG: photoplethysmography
PR: pulse rate
PRISMA: Preferred Reporting Items for Systematic Reviews and Meta-Analyses
RESTORE2: Recognise Early Soft Signs, Take Observations, Respond, Escalate
rpm: respirations per minute
rPPG: remote photoplethysmography
RR: respiratory rate
SBP: systolic blood pressure
SOC: standard of care
VISION: Vital Sign Comparison Between Lifelight and Standard of Care
VISION-D: Vital Sign Comparison Between Lifelight and Standard of Care: Development
VISION-MD: Vital Sign Comparison Between Lifelight and Standard of Care: Multisite Development
VISION-V: Vital Sign Comparison Between Lifelight and Standard of Care: Validation
VS: vital sign
